# The Impact of Thermal and Electrical Pretreatments and Antibrowning Solution on the Chlorogenic and Dicaffeoylquinic Acid Extraction Yield from Endive Roots

**DOI:** 10.3390/molecules30102091

**Published:** 2025-05-08

**Authors:** Etienne Diemer, Morad Chadni, Irina Ioannou, Nabil Grimi

**Affiliations:** 1 URD Agro-Biotechnologies Industrielles (ABI), CEBB, AgroParisTech, 51110 Pomacle, France; etienne.diemer@gmail.com (E.D.); i.ioannou@groupe-esa.com (I.I.); 2 Centre de Recherche Royallieu—CS 60319, Transformations Intégrées de la Matière Renouvelable (TIMR), Université de Technologie de Compiègne UTC/ESCOM, CEDEX, 60203 Compiègne, France

**Keywords:** caffeoylquinic acids, pulsed electrical field, microwave, pressing, oxidation, forced endive roots

## Abstract

Forced endive roots (FERs) contain beneficial antioxidant compounds such as chlorogenic acid (5-CQA) and dicaffeoylquinic acids (diCQAs). This study compared the extraction yields of 5-CQA and diCQAs using a biomass pressing method with various pretreatments, including pulsed electric field (PEF) and microwave (MW), against the solid–liquid extraction method (water, 90 °C, 30 min). The results indicated that the MW pretreatment achieved the highest yields, extracting 28 ± 2% of 5-CQA and 13 ± 1% of diCQAs, surpassing the solid–liquid method. Furthermore, the oxidative degradation of CQAs was studied, and it appeared that this reaction was enhanced by PEF pretreatment. An antibrowning solution (ABS) was successfully tested to reduce this oxidation and protect CQAs. An extraction process utilizing MW and PEF pretreatments combined with an ABS solution achieved yields of 65 ± 1% for diCQAs and 80 ± 5% for 5-CQA, significantly outperforming the solid–liquid extraction method.

## 1. Introduction

The Belgian endive (*Cichorium intybus* L.) is widely cultivated across Europe, with France producing approximately 160,000 tons annually. Notably, 95% of this production occurs in the Hauts-de-France region. The cultivation of Belgian endives, also referred to as chicons, involves a two-stage process. In the first stage, the roots are grown in fields and harvested. During the second stage, these roots undergo a forcing process in complete darkness for 21 days at temperatures between 16 and 20 °C, resulting in the formation of chicons.

After the chicons are harvested, the forced endive roots (FERs), a by-product of this process, are left behind. FERs are typically used in low-value applications, such as livestock feed supplements, composting, or as field amendments. Since one root is produced for each chicon, FERs constitute the primary by-product of Belgian endive cultivation.

Despite their current uses, FERs hold significant potential due to their richness in bioactive compounds, particularly phenolic compounds known for their health-promoting properties. Specifically, FERs are abundant in chlorogenic acid (5-CQA) and dicaffeoylquinic acids (diCQAs) [1,2,3,4], which are valuable for their antioxidant and anti-inflammatory benefits. The extraction of CQAs from FERs represents an essential step in the valorization of this by-product, offering opportunities to enhance its economic and functional value within the framework of sustainable agriculture and circular economy practices.

Traditionally, CQAs have been extracted using solid–liquid extraction methods, which involve dissolving the desired compounds in a solvent and separating them from the solid plant material. This technique, though effective, has certain limitations in terms of efficiency and sustainability, particularly with respect to solvent use and energy consumption [5,6]. Over the years, various studies have focused on optimizing the extraction of CQAs using conventional methods, such as adjusting solvent type, temperature, and extraction time [7,8]. Additionally, innovative techniques such as microwave-assisted extraction [9] and ultrasound-assisted extraction [10,11,12] have emerged as alternatives, offering the potential to improve extraction efficiency and reduce extraction times.

In recent years, the use of supercritical fluids, especially supercritical carbon dioxide (CO_2_), has gained attention as a promising method for 5-CQA extraction. This approach offers advantages such as selective extraction, high extraction yields, and environmentally friendly processing [13]. Despite these advances, traditional thermal methods, including those based on diffusion, still have several drawbacks. These include high energy consumption, the potential for juice dilution, and a reduction in the quality of the final product. Thermal treatments may also lead to the degradation of sensitive compounds, such as polyphenols, affecting the overall yield and bioactivity of the extracted substances.

Another significant technique for the recovery of bioactive compounds, especially water-soluble ones such as sugars and inulin, is biomass pressing. This method is widely employed in industries where the extraction of soluble compounds from plant biomass is required. Several studies have investigated the extraction of polyphenols and other bioactive molecules by pressing, demonstrating its efficiency in recovering valuable compounds from plant material [14,15,16,17,18]. However, to maximize extraction yields and improve the quality of the extracted compounds, pretreatment of the biomass is often necessary. Pretreatment methods can help break down cell walls, release compounds of interest, and optimize extraction efficiency.

The most commonly used pretreatment techniques include thermal treatments, such as microwave pretreatment [19,20,21,22], enzymatic-assisted extraction [23], and electrical treatments such as pulsed electric field (PEF) [24,25]. These methods aim to enhance the extraction process by improving the accessibility of the target compounds within the plant tissue. However, while these techniques have shown promise, further research is required to fully understand the specific effects of thermal and electric fields on the recovery of CQAs from plant materials.

A major concern in the extraction of CQAs is the potential for oxidation, which can occur during both the pressing and pretreatment stages of extraction. CQAs are particularly susceptible to enzymatic oxidation, leading to the degradation of the compounds and a reduction in their bioactivity [26,27]. Therefore, it is essential to control oxidative processes during extraction to maintain the integrity of the extracted CQAs. Studies have shown that the use of antioxidative solutions or inhibitors, such as an antibrowning solution (ABS), can effectively mitigate oxidation during pressing and pretreatment, preserving the quality of the extracted compounds [28].

To the best of our knowledge, the optimization of process parameters for the extraction of CQAs from forced endive roots (FERs) using pressing in combination with PEF or MW pretreatments has not been extensively studied. These techniques, when combined, have the potential to significantly reduce energy consumption and process time compared to traditional methods, offering a more efficient and sustainable alternative for the extraction of valuable bioactive compounds. The objective of this study was to explore the potential of PEF and MW pretreatments applied to FERs in order to improve both the extraction yield and recovery of CQAs. In addition to assessing the pressing kinetics and juice characteristics, such as °Brix (soluble solids content), special attention was given to investigating the oxidative effects that may occur during the pretreatment and pressing stages. Furthermore, the study sought to evaluate the effectiveness of an antibrowning solution (ABS) in preventing oxidation and enhancing the overall quality of the extracted CQAs.

## 2. Results and Discussion

### 2.1. Extracted Yield

#### 2.1.1. Effect of Cossette Size on Extracted Yield

**Figure 1:**
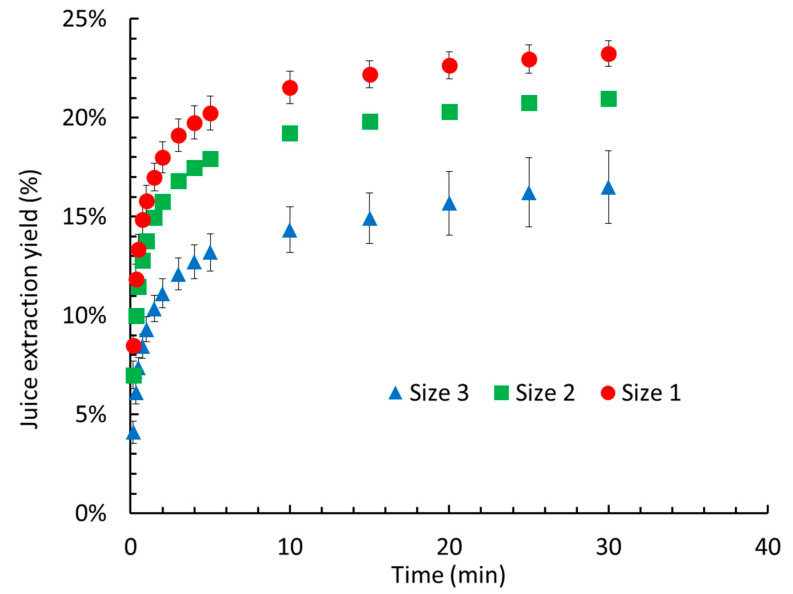
Extraction yield kinetics for different cossette sizes under 3 bar pressing.

The size of the cossettes has a significant impact on juice release; thinner cossettes offer less resistance, thereby facilitating juice extraction [29]. The effect of cossette size on the extracted yield is illustrated in Figure 1 (Table S1).

A smaller cossette size was associated with a higher extraction yield. For instance, after 30 min of pressing, the yield for the smallest size (size 1) was 6% higher than that for size 3. A similar trend has been observed in apples and carrots [25]. However, because of the minimal difference in yield between size 1 and size 2, size 2 was selected for subsequent experiments.

#### 2.1.2. Effect of Working Pressure on Extracted Yield

**Figure 2:**
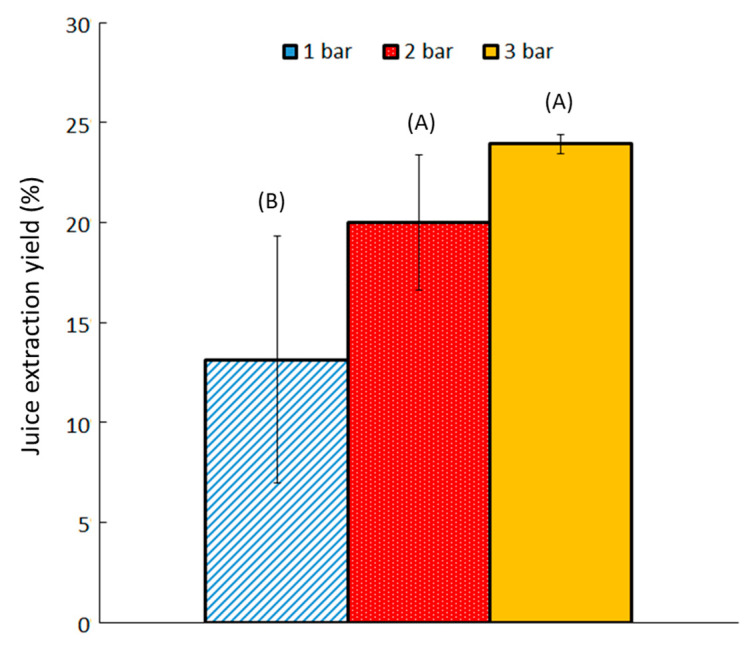
Yield obtained at varying working pressures after 30 min of pressurization using cossette size 2. Bars with different letters indicate significant differences in the yield of extracted juice (*p* > 0.05) according to the Tukey test at 95%.

The impact of working pressure on the extracted yield was also investigated (Figure 2). Three different pressures were applied: 1 bar, 2 bar, and 3 bar.

Extracted yield increased with increasing working pressure. Increasing the pressure from 1 to 3 bar enhanced the extracted yield from 13 ± 1% to 24 ± 1%. The same effect of pressure has been observed for chicory roots [29]. A working pressure of 3 bar was used for subsequent experiments. The yield remained low; incorporating pretreatment steps before pressing might enhance it. Cell denaturation, in particular, seems to be a promising approach for increasing the extraction yield.

#### 2.1.3. Effect of Pretreatment on Extracted Yield

**Figure 3:**
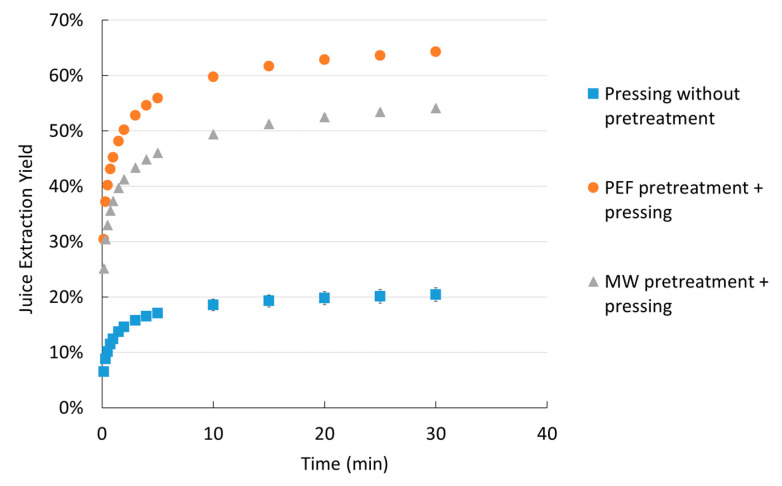
Effects of MW and PEF pretreatments on extracted yield kinetics.

The effects of PEF and MW pretreatments prior to pressing are shown in Figure 3. A comparison with pressing without pretreatment was also conducted.

Figure 3 shows that both pretreatments had a significant effect, resulting in a much higher extracted yield. This improved yield can be attributed to cell membrane degradation, which occurs with MW when temperatures exceed 60 °C because of thermal treatment caused by liquid expansion [30,31] or with PEF through the electropermeabilization of cells [32]. However, PEF pretreatment leads to better cell denaturation than microwave pretreatment, with lower energy consumption (40 kJ/kg and 600 kJ/kg, respectively). For example, after 30 min of pressing, only a 20 ± 1% extracted yield was obtained without pretreatment, while PEF and MW pretreatments resulted in yields of 64 ± 1% and 54 ± 1%, respectively. Thus, the extracted yield was more than three times higher with PEF pretreatment and two and a half times higher with MW pretreatment.

### 2.2. Juice Characterization

#### 2.2.1. Total Soluble Matter (°Brix)

The soluble matter content in the juice primarily comprised sugars and organic acids, which are predominantly found in the vacuole sap. MW or PEF pretreatments promote the effective breakdown of intact cells and vacuoles, facilitating the release of juice and subsequent extraction of the vacuole sap [30,33,34]. Soluble matter values in °Brix are listed in Table 1.

Compared with the untreated samples, both the PEF- and MW-pretreated samples exhibited statistical increases (*p* < 0.05) in soluble matter content. A comparable trend was observed when PEF-assisted pressing was applied to the extraction of carrot [29]. Increasing temperature using conventional solid–liquid extraction of sugar beets also led to an increase in the soluble matter content [34].

#### 2.2.2. CQA Content

**Figure 4:**
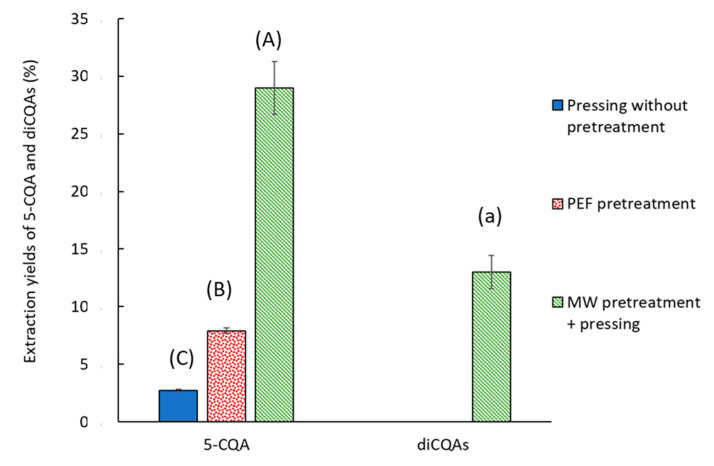
CQA extraction yields with different pretreatment conditions. The extraction yield is given relative to a conventional solid–liquid extraction. Bars with different small letters indicate significant differences in the yield of diCQAs (*p* > 0.05) according to the Tukey test at 95%. Bars with different capital letters indicate significant differences in the yield of 5-CQA.

The extraction yields of 5-CQA and diCQAs compared with conventional solid–liquid extraction are displayed in Figure 4.

MW pretreatment resulted in the extraction of approximately 29 ± 2% of 5-CQA and 13 ± 2% of diCQAs, whereas untreated and PEF-pretreated pressing extracts yielded less than 8% of 5-CQA and no diCQAs. The primary hypothesis is that interactions between CQAs and the biomass hindered extraction. These interactions can be disrupted only by thermal treatment [19,35]. Numerous studies have reported interactions between cell wall polysaccharides and chlorogenic acid in apples and cellulose–pectin composites [36,37]. Hydrogen bonds, hydrophobic interactions, and electrostatic interactions between chlorogenic acid and cellular structures have also been documented. Investigating the effect of thermal pretreatment duration on CQA extraction is essential to optimize energy consumption during MW pretreatment. PEF pretreatment did not allow efficient extraction of CQAs, perhaps because of its limited ability to completely disrupt cellular structures such as vacuoles and rigid cell walls where these compounds are stored. In addition, PEF does not generate heat, which is essential to solubilize and release bound phenolics. As a result, only slight improvements were observed for 5-CQA, and no extraction occurred for diCQAs. This highlights the limited efficacy of PEF for recovering these compounds.

### 2.3. Effect of Thermal Pretreatment

**Figure 5:**
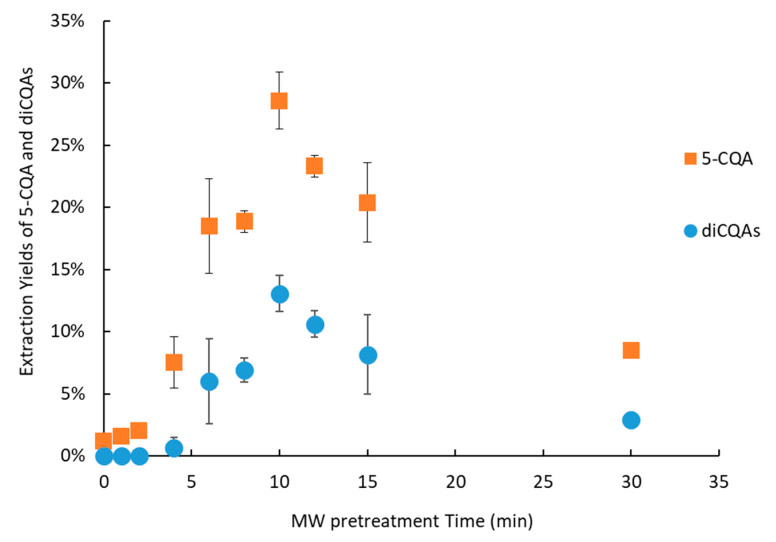
Extraction yields of 5-CQA and diCQAs with different MW pretreatment times. The extraction yield is given relative to a conventional solid–liquid extraction.

The MW pretreatment time before pressing was studied. Results are shown in Figure 5 (Table S2).

**Figure 6:**
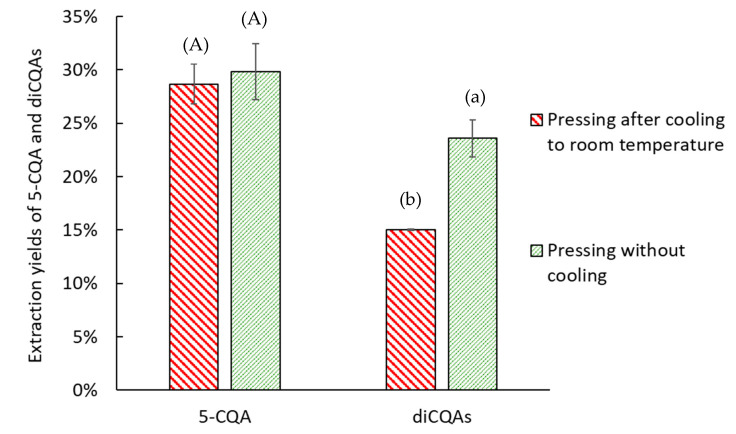
Effect of FER cooling on CQA extraction yield. The extraction yield is given relative to a conventional solid–liquid extraction. Bars with different small letters indicate significant differences in the yield of diCQAs (*p* > 0.05) according to the Tukey test at 95%. Bars with different capital letters indicate significant differences in the yield of 5-CQA.

The optimal MW pretreatment time was 10 min for both 5-CQA and diCQAs, yielding 29 ± 2% for 5-CQA and 13 ± 2% for diCQAs. After 10 min of MW treatment, a temperature of 100 °C was reached (Figure S1), consistently with the literature [35]. After 10 min, the extracted yield decreased because of water evaporation during MW treatment, which in turn reduced the CQA yield. The yield of diCQAs was half that of 5-CQA, which can be attributed to the double caffeic acid moiety, a highly hydrophobic component. This part of the CQAs interacts with the cell wall through noncovalent bonding, which likely limits its extraction [36,38]. To estimate the readsorption of CQAs on the FER cell wall during cooling, pressing was conducted immediately after 10 min of MW pretreatment and after cooling to room temperature. The results are shown in Figure 6.

During cooling to room temperature, some of the diCQAs appeared to readsorb onto the cell wall, making them less accessible for extraction by pressing. As a result, 5-CQA seemsed to be less affected, with no statistically significant difference in extraction yield (*p* > 0.05). This was likely due to its better water solubility compared with diCQAs [39]. While pressing with MW pretreatment enhanced CQA extraction, a threshold appeared to be attained with pressing. Consequently, it seems imperative to introduce a solvent to enhance the recovery of a larger quantity of CQAs. Water was chosen as a green and inexpensive solvent.

### 2.4. Effect of Water Addition

**Figure 7:**
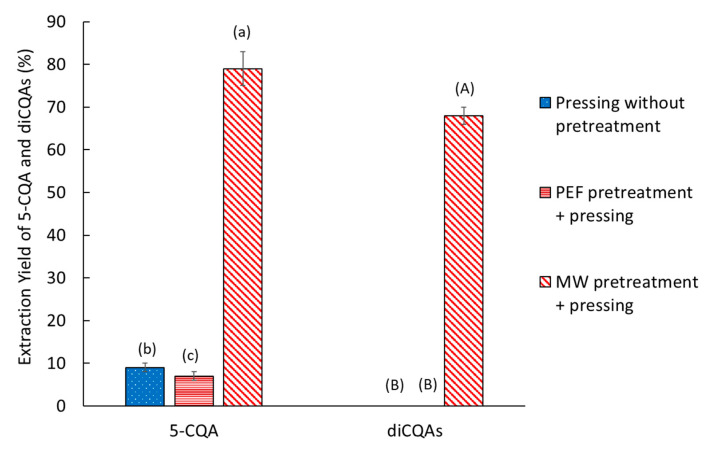
Effect of water addition on CQA extraction yield with different pretreatment conditions. The extraction yield is given relative to a conventional solid–liquid extraction. Bars with different small letters indicate significant differences in the yield of 5-CQA (*p* > 0.05) according to the Tukey test at 95%. Bars with different capital letters indicate significant differences in the yield of diCQAs.

The main objective was to increase yield while maintaining ecoefficient extraction. The effect of water addition on pressing, assisted by pretreatment, was measured (Figure 7). Water was added to the FERs at a 1/1 ratio of fresh weight to liquid immediately after cutting.

The addition of water to a MW pretreatment significantly increased the extraction of 5-CQA (from 30 ± 2% to 79 ± 4%) and diCQAs (from 24 ± 2% to 68 ± 2%) compared with extraction without solvent. In addition, pressing alone with the addition of solvent improved the extraction of 5-CQA from 2 ± 1% to 9 ± 1%. The addition of water seems to help the diffusion of the CQAs in the extraction medium and to limit the readsorption of the CQAs by the plant material, explaining the improved yields. However, for diCQAs, extraction by pressing without pretreatment showed no improvement in yield. Similarly, the use of pretreatment by PEF did not bring any significant benefit to the extraction efficiency of CQAs compared with solvent-free extraction. Heat treatment seems necessary to desorb the CQAs. Moreover, low yields with PEF can be caused by oxidation of CQAs by polyphenol oxidase (PPO), which leads to their degradation into brown components (allomelanins). In our previous work [40], this oxidation was assumed to occur during drying, but it also seems to occur during pretreatments and pressing [41,42]. An antibrowning solution (ABS) can be used to prevent this oxidation [43].

### 2.5. Effect of PEF + ABS Pretreatment on FER CQA Content

**Figure 8:**
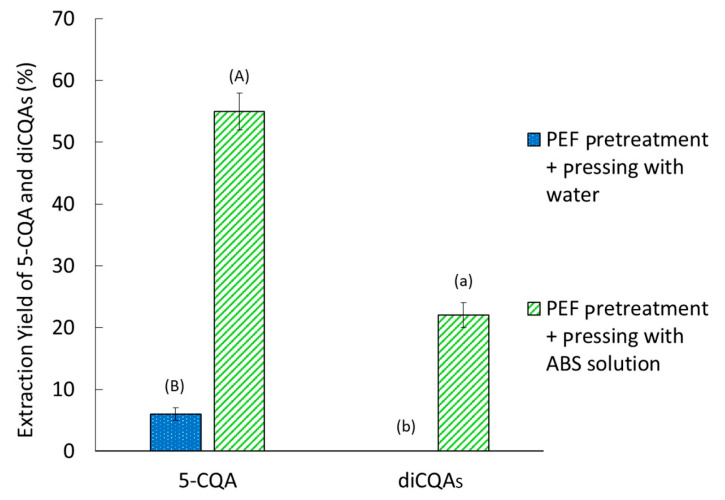
Effect of an antibrowning solution or water addition on CQAs extraction yield. The extraction yield is given relative to a conventional solid–liquid extraction. Bars with different small letters indicate significant differences in the yield of diCQAs (*p* > 0.05) according to the Tukey test at 95%. Bars with different capital letters indicate significant differences in the yield of 5-CQA.

The effect of the antioxidant solution on extracted CQA yields was studied (Figure 8). Water or the antioxidant solution was added to the FERs at the same ratio as before after cutting. The antioxidant solution was made by ascorbic acid (1%) and oxalic acid (0.02%).

The ABS solution boosted the CQA extraction, increasing diCQA extraction from 0 to 22% and 5-CQA extraction from 6% to 55%. ABS treatment appears to prevent PPO activity and CQAs degradation after PEF pretreatment. Thus, the partial release of CQAs can be achieved by electric treatment to disrupt the weak interactions between CQAs and cell wall polysaccharides. With ABS treatment, FERs were brighter even after PEF pretreatment, with a less visible brown part (Figure S2). Browning prevention is an indication of PPO activity prevention and preservation of CQAs [43].

### 2.6. Coupling Pretreatments and Antibrowning Treatment

**Figure 9:**
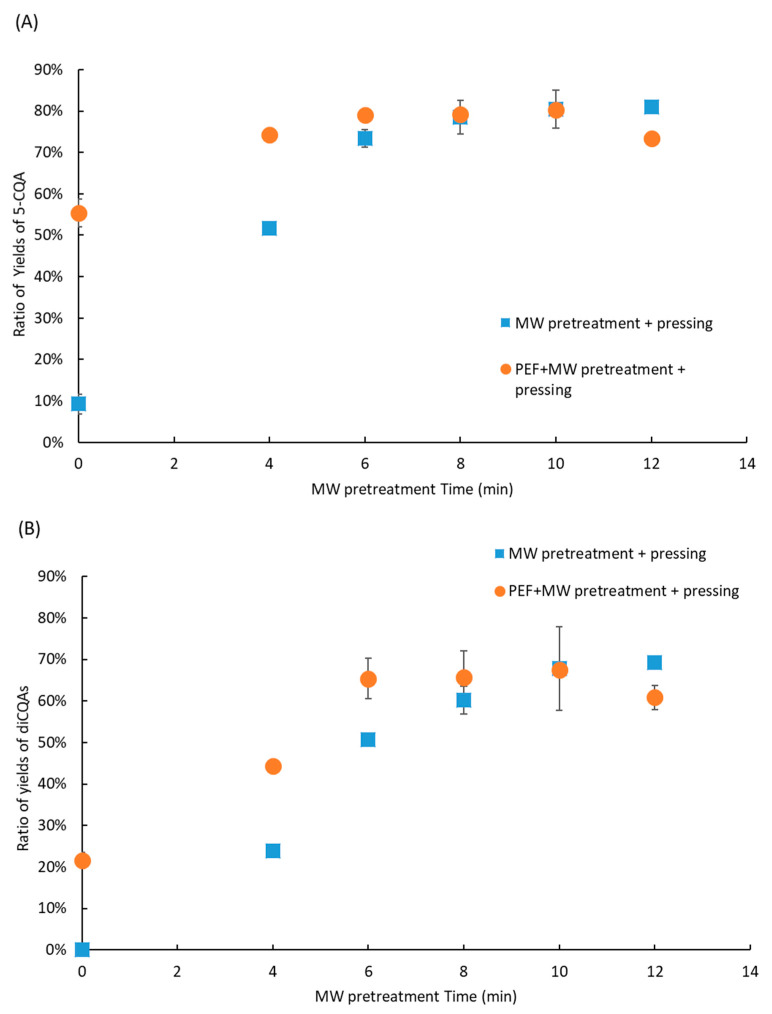
Effect of PEF and MW pretreatments on 5-CQA (**A**) and diCQA (**B**) extraction yield, with FERs treated with an ABS solution prior to pretreatment.

The CQA yield is limited by the extracted yield, adsorption of CQAs onto the FER cell wall, and PPO degradation. To overcome these limitations, MW and PEF pretreatments can be coupled with pressing to increase CQA yield. The results are presented in Figure 9A,B (Table S3). FERs were mixed with the ABS before any pretreatment. In all pretreatment conditions, including PEF, microwave, and their combination, the antibrowning solution was used systematically to limit enzymatic degradation of phenolic compounds. A comparison without prior PEF pretreatment was also conducted to assess the effect of adding PEF on extraction.

As observed previously, the yield of diCQAs was lower than that of 5-CQA. However, the yields of 5-CQA and diCQAs with coupled pretreatments were significantly higher than those obtained with only one pretreatment up to 6 min of MW pretreatment. This increase can be attributed to the cell permeabilization effect caused by PEF, which allows CQAs to be released more easily into the medium. The combination of PEF and MW pretreatments did not improve the maximum CQA yield, but it helped achieve the maximum yield with a reduced MW pretreatment duration, from 10 to 6 min. PEF pretreatment also reduced energy consumption (from 600 kJ/kg to 400 kJ/kg) while achieving optimal extraction yield. This could be explained by the fact that PEF-induced membrane permeabilization may enhance the release of CQAs but make them more susceptible to thermal degradation under extended microwave exposure.

## 3. Materials and Methods

### 3.1. Raw Materials

Forced Witloof Belgian endive roots (FERs) (*Cichorium intybus* L.) of the cultivars ‘Flexine–Vilmorin’ and ‘Laurine’ were provided by the Association des Producteurs d’Endive de France’s experimental station in Arras, France. The FERs were washed with cold tap water and stored in the dark at 4 °C until further use. They were then cut into cossettes using food-cutting equipment (Robot Coupe CL50, S.N.C, Vincennes, France). Three sizes of cossettes were prepared: S^1^ (2 × 2 × 55 mm^3^), S^2^ (2 × 5 × 55 mm^3^), and S^3^ (3 × 5 × 55 mm^3^). After cutting, the FERs were mixed and randomly distributed across each experimental condition.

### 3.2. Analytical Reagents and Chemicals

Acetonitrile (99.9%), ethanol (99.9%), ascorbic acid (99%), and formic acid (98–100%) were purchased from Thermo Fisher Scientific (Illkirch, France). Methanol (99%) and oxalic acid (technical grade) were obtained from VWR (Fontenay-Sous-Bois, France). Ultrapure water was produced using a Milli-Q system (Millipore Corporation, Burlington, MA, USA). Chlorogenic acid standard (5-CQA) (>98%) was supplied by Sigma (Saint-Quentin-Fallavier, France), while 3,5-dicaffeoylquinic acid (3,5-diCQA), 3,4-dicaffeoylquinic acid (3,4-diCQA), and 4,5-dicaffeoylquinic acid (4,5-diCQA) were obtained from Carbosynth (Compton, UK).

### 3.3. Extraction Procedure

**Figure 10:**
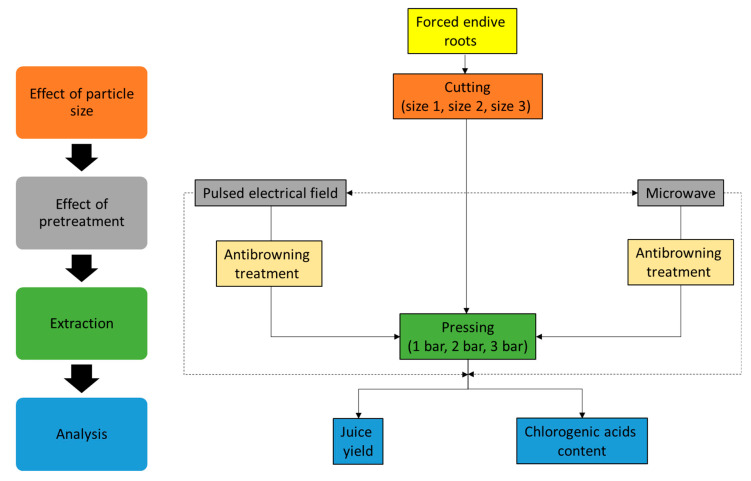
Experimental process scheme.

FERs were cut into cossettes, and the impact of different physical (PEF, MW, cutting size) and chemical (ABS) pretreatments on the extraction yield and CQA yield was evaluated. The experimental procedure is summarized in Figure 10. Approximately 500 g of FERs were used for each experimental condition.

#### 3.3.1. Pressing Experiments

A laboratory press chamber (hemispherical in shape, with a radius of R = 114 mm and a surface area of A = 817 cm^2^) equipped with an elastic diaphragm was used for the pressing experiments. The FER cossettes were pressed at 3 bar, except in Section 2.1.2. The extracted juice was regularly weighed using a balance (Sartorius Entris II, Göttingen, Germany) to generate a pressing kinetic curve.

As previously described [38], the extracted yield $Y_{i}$ was calculated as follows:

(1) $$Y_{i}\left(\%\right)=\frac{m_{juice}}{m_{FER}}\times 100$$

where $m_{juice}$ is the mass of extracted juice during pressing (g) and $m_{FER}$ is the initial mass of the FERs before pressing (g).

#### 3.3.2. Diffusion Experiments

As previously described [38], the conventional solid–liquid extractions were performed for 30 min in a 500 mL Erlenmeyer flask covered with aluminum foil to prevent water loss. Water was used as the solvent. The extractions were conducted at 90 °C with a solid–liquid ratio of 1:100 to extract all CQAs. In all experiments, the agitation speed was maintained at 400 rpm.

### 3.4. Physical and Chemical Pretreatments

#### 3.4.1. Pulsed Electrical Field Pretreatment

For the PEF treatment of cossettes, a pilot PEF generator (5 kV–1 kA; Hazemeyer, Saint-Quentin, France) was used. The treatment chamber consisted of two flat stainless-steel electrodes (230 mm × 265 mm), which generated near-rectangular monopolar pulses.

The distance between the electrodes was 8 cm. Cossettes were placed between the electrodes either without any solvent (for Section 3.1 and Section 3.2) or with solvent (for Section 3.3, Section 3.4, Section 3.5) at a solid/liquid ratio of 1:1.

A fixed electric field strength of 600 V·cm^−1^ was used for all the experiments. The samples were PEF-treated with N = 20 trains of impulsions. Each train consisted of n = 100 pulses, with a pulse duration of t_i_ = 100 μs and a rest time of 10 s after each train of impulsions. The total time of PEF treatment was calculated as follows:

(2) $$t_{PEF}\left(s\right)=n\times N\times t$$

with t_PEF_ fixed at 0.2 s. The temperature of the FERs did not rise by more than 5 °C during treatment (maximum of 25 °C after PEF treatment).

#### 3.4.2. Microwave Pretreatment

The MW treatment was performed using a Milestone EOS-G microwave laboratory oven (Sorisole, Bergamo, Italy). This device is a multimode microwave reactor operating at 2.45 GHz with 10 W increments and a maximum power output of 900 W. FER cossettes were heated at a constant power density of 1 W/g. An optical fiber was used to monitor the temperature inside the plant material.

Cossettes were either cooled to room temperature before pressing or directly used for pressing experiments, as described in Section 3.5. The initial temperature of the FER was 22 °C, rising to 100 °C after 10 min of MW treatment (Figure S1, supplementary data).

#### 3.4.3. Antibrowning Treatment

A solution containing 1% (*w*/*w*) ascorbic acid and 0.2% (*w*/*w*) oxalic acid was prepared, as described by Son et al. (2001) [44]. This solution is referred to as ABS. FER cossettes were immersed in the solution for 10 min at a solid–liquid ratio of 1:1. Subsequently, the cossette–solution mixture was used for the pretreatment and pressing stages of the extraction process described in Section 3.3 and Section 3.5.

### 3.5. Analytical Measurements

#### 3.5.1. Soluble Matter

The total soluble matter (°Brix) in the juice was measured using an AR digital refractometer (Leica Microsystems Inc., Buffalo, NY, USA).

#### 3.5.2. HPLC Analysis

Quantification of CQAs was performed as previously described [38]. The identification and quantification of 5-CQA, 3,5-diCQA, 3,4-diCQA, and 4,5-diCQA content were achieved by comparing their relative retention times with those of the corresponding standard compounds. The sum of 3,5-diCQA, 3,4-diCQA, and 4,5-diCQA was considered as dicaffeoylquinic acids (diCQAs).

### 3.6. CQA Extraction

In this study, the extraction ratios of 5-CQA and diCQAs, in terms of yield compared with solid–liquid extraction, were calculated as follows:

(3) $$Y_{5-CQA}\left(\%\right)=\frac{m_{5-CQApressing}}{m_{5-CQAS:L}}*100$$

(4) $$Y_{diCQAs}\left(\%\right)=\frac{m_{diCQAspressing}}{m_{diCQAsS:L}}*100$$

where, $m_{5-CQApressing}$ and $m_{diCQAspressing}$ were the masses (g) of 5-CQA and diCQAs, respectively, extracted by pressing with or without pretreatment. $m_{5-CQAS:L}$ (g) and $m_{diCQAsS:L}$ were the masses of 5-CQA and diCQA, respectively, extracted by the solid–liquid extraction presented before. Y_molecule_ refers to either Y_5-CQA_ or Y_diCQAs_.

In this work, solid–liquid extraction was used as the reference method to determine the total CQA content, as previously investigated in our studies [1]. Using a 100:1 solvent-to-solid ratio, this method yielded the highest amounts of 5-CQA and di-CQA. While the pressing process can lead to higher concentrations of these compounds in the extract, the overall recovery remains lower than that achieved through solid–liquid extraction.

### 3.7. Energy Consumption Measurement

The energy consumption of the PEF treatment (*W*, J/kg) was calculated using the following formula:

(5) $$W=(U\times I\times t_{p})/m$$

where *U* is the PEF voltage (V), *I* is the current intensity (A), *t_P_* is the total pulse duration (s), and m is the total mass of treated sample (kg).

For the MW treatment, the power was fixed for the experiment. Thus, as previously described [38], the energy consumption was calculated as follows:

(6) $$E\left(kJ/kg\right)=\frac{P\times t}{m}$$

where E is the energy consumption (kJ/kg fresh weight), P is the power of the MW or PEF, t is the time (s), and m is the mass of fresh FERs (kg).

### 3.8. Statistical Analyses

All extractions were performed at least in duplicate. The average values and standard errors were calculated with Excel, 2016 version. Significant differences (*p* < 0.05) between samples were determined using ANOVA. A Tukey test was applied to identify significant differences between groups, represented by different letters in the figures.

## 4. Conclusions

This study highlights the significant impact of thermal and electrical pretreatments on juice extraction and the recovery of chlorogenic acids (CQAs) from FERs. The application of pulsed electric field (PEF) pretreatment resulted in a higher extraction yield compared with microwave (MW) pretreatment, demonstrating the superior cell membrane permeabilization effect of the electrical field over thermal treatment. However, the recovery of CQAs was higher with MW pretreatment, suggesting that thermal treatment plays a critical role in desorbing CQAs from the plant cell wall matrix, thereby facilitating their release into the juice.

An interesting observation was the oxidative effect associated with pressing and extraction, which was amplified by PEF treatment, leading to CQA degradation. In contrast, treatment with aqueous biphasic systems (ABSs) effectively mitigated oxidative degradation, significantly enhancing CQA recovery from the biomass.

The combined use of MW, PEF, and ABS pretreatments before pressing produced outstanding results, achieving increased recovery rates of 65% diCQAs and 80% 5-CQA compared with conventional solid–liquid extraction. This innovative approach not only yielded highly concentrated juice but reduced energy consumption. The extracted juice could serve as a precursor for the production of high-value bioactive compounds, underscoring the potential of this method for industrial applications.

## Figures and Tables

**Table 1:** Characteristics of FER juice obtained from different pretreatment methods. * for statistical difference (*p* < 0.05) with pressing alone.

Condition	Soluble Matter (°Brix)
Pressing without pretreatment	6.0 ± 0.0
PEF pretreatment + pressing	7.5 ± 0.6 *
MW pretreatement + pressing	9.3 ± 0.6 *

## Data Availability

The original contributions presented in this study are included in the article/Supplementary Material. Further inquiries can be directed to the corresponding authors.

## Supplementary Materials

The following supporting information can be downloaded at: https://www.mdpi.com/article/10.3390/molecules30102091/s1, Figure S1: Microwave heating curve; Figure S2: With ABS (left picture) and without ABS (right picture); Table S1: Extraction yield kinetics for different cossette sizes under 3 bar pressing; Table S2: 5-CQA and diCQAs extraction yield with different MW pretreatment time. The extraction yield is given relative to a conventional solid-liquid extraction; Table S3: Effect of PEF and MW Pretreatments on 5-CQA and diCQAs Extraction Yield, with FER treated with an ABS Solution prior to pretreatment.

## Abbreviations

5-CQA, chlorogenic acid; ABS, antibrowning solution; diCQAs, dicaffeoylquinic acids; FERs, forced endive roots; MW, microwave; PEF, pulsed electrical field; PPO, polyphenol oxidase.
