# Supplementary material for: The Impact of Thermal and Electrical Pretreatments and Antibrowning Solution on the Chlorogenic and Dicaffeoylquinic Acid Extraction Yield from Endive Roots
Source: Molecules. 2025 May 8;30(10):2091. doi: 10.3390/molecules30102091 (PMC12114360; doi:10.3390/molecules30102091)
Supplement: Supplementary file 1 [file molecules-30-02091-s001.zip › molecules-3515874-supplementary.pdf]

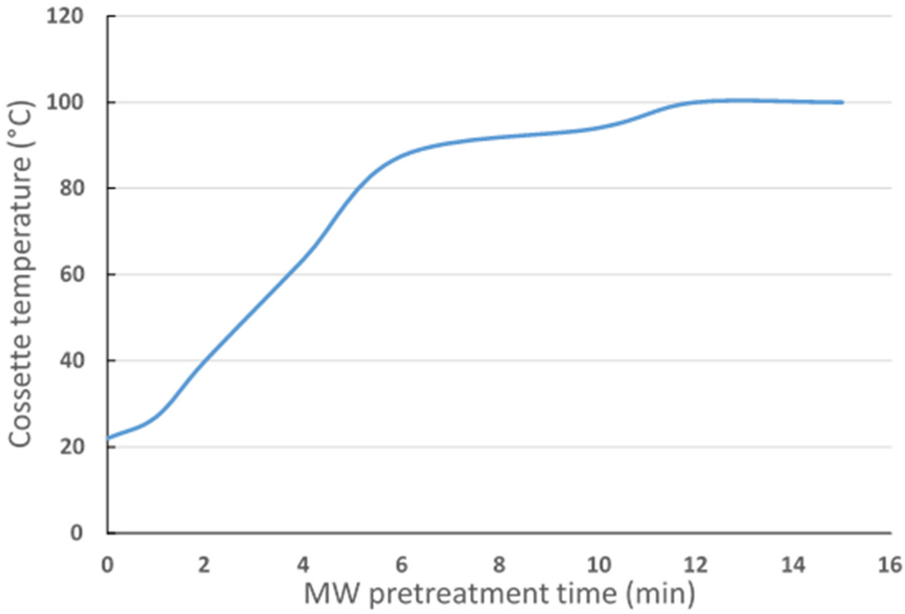

Figure S1 : MW heating curve

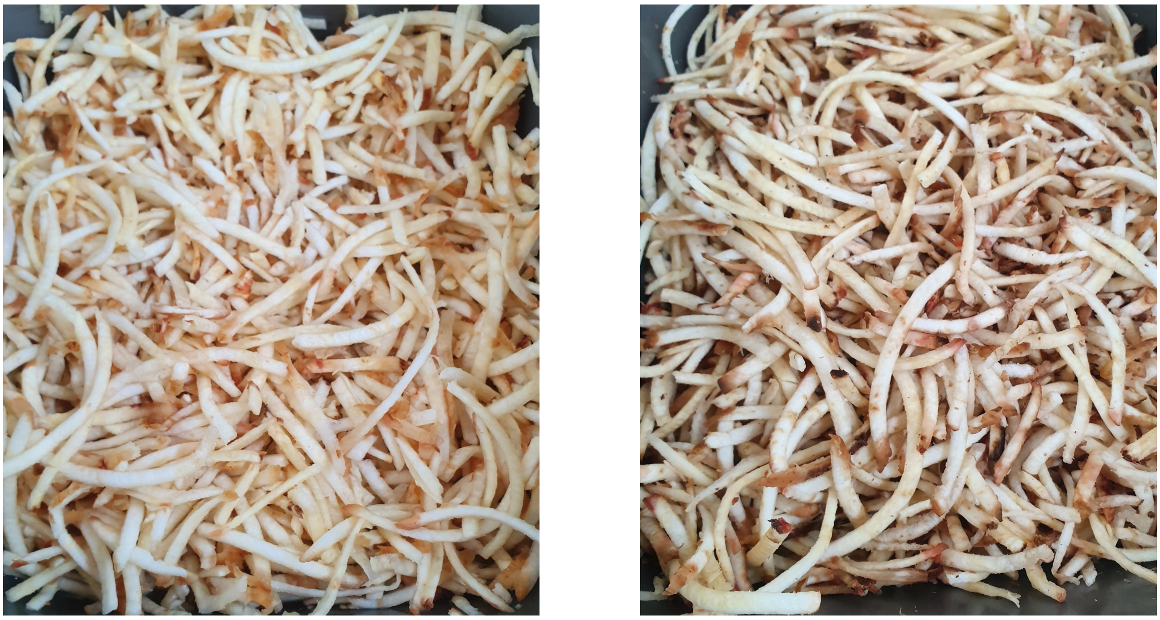

Figure S2: With ABS (left picture) and without ABS (right picture)

Table S1: Extraction yield kinetics for different cossette sizes under 3 bar pressing.

	Juice Extraction Yield (%)		
Time (min)	Size 3	Size 2	Size 1
0.166666667	4±0.57	7±0.71	9±0.49
0.333333333	6±0.57	10±0.14	12±0.74
0.5	7±0.53	11±0.11	13±0.74
0.75	8±0.60	13±0.14	15±0.81
1	9±0.64	14±0.18	16±0.78
1.5	10±0.67	15±0.04	17±0.71
2	11±0.72	16±0.04	18±0.78
3	12±0.81	17±0.01	19±0.83
4	13±0.86	18±0.01	20±0.84
5	13±0.94	18±0.00	20±0.86
10	14±1.16	19±0.00	22±0.81
15	15±1.27	20±0.03	22±0.70
20	16±1.61	20±0.01	23±0.68
25	16±1.74	21±0.03	23±0.73
30	17±1.84	21±0.06	23±0.65

Table S2: Extraction yields of 5-CQA and diCQAs with different MW pretreatment times. The extraction yield is given relative to a conventional solid–liquid extraction.

	Extraction Yield (%)	
Time (min)	5-CQA	diCQAs
0	1±0.17	0±0
1	2±0.08	0±0
2	2±0.25	0±0
4	8±2.06	1±0.88
6	18±3.80	6±3.41
8	18.8±0.8	6.9±1.0
10	29±2.29	13±1.45
12	23.3±0.9	10.6±1.1
15	20±3.19	8±3.21
30	9±0.33	3±0.12

Table S3: Effects of PEF and MW pretreatments on 5-CQA and diCQA extraction yields, with FERs treated with an ABS solution prior to pretreatment.

	5-CQA Yield (%)		diCQA Yield (%)	
Time (min)	MW+pressing	PEF+MW+pressing	MW+pressing	PEF+MW+pressing
0	9±2.4	55±3.3	0±0	22±2.0
4	52±0.0	74±0.6	24±0.8	44±0.3
6	73±2.2	79±0.2	51±1.0	65±4.8
8	79±4.1	79±1.2	60±3.4	66±6.4
10	80±4.6	80±0.4	68±10.1	67±1.0
12	81±1.0	73±0.2	69±0.4	61±2.9
